# Implementation research on sustainable electrification of rural primary care facilities in Ghana and Uganda

**DOI:** 10.1093/heapol/czaa077

**Published:** 2020-11-06

**Authors:** Dena Javadi, John Ssempebwa, John Bosco Isunju, Lucy Yevoo, Alberta Amu, Elizabeth Nabiwemba, Michaela Pfeiffer, Irene Agyepong, Luc Severi

**Affiliations:** 1 Alliance for Health Policy and Systems Research, World Health Organization, 20 Ave Appia, Geneva 1211, Switzerland; 2 Department of Disease Control and Environmental Health, Makerere University School of Public Health, New Mulago Hill Road, Kampala, Uganda; 3 Ghana Health Service, Dodowa Health Research Center, Hospital Road, Matetse, Ghana; 4 Department of Community Health and Behavioural Science, Makerere University School of Public Health, New Mulago Hill Road, Kampala, Uganda; 5 Department of Public Health Environmental and Social Determinants of Health, World Health Organization, 20 Ave Appia, Geneva 1211, Switzerland; 6 Ghana College of Physicians and Surgeons, Public Health Department, 54 independence Ave, Accra, Ghana; 7 Sustainable Energy for All, 1750 Pennsylvania Ave, Washington, DC, USA

**Keywords:** Sustainable energy, implementation research, integration, availability and readiness, Low-and Middle-Income Countries (LMIC), Uganda, Ghana

## Abstract

Access to energy is essential for resilient health systems; however, strengthening energy infrastructure in rural health facilities remains a challenge. In 2015–19, ‘Powering Healthcare’ deployed solar energy solutions to off-grid rural health facilities in Ghana and Uganda to improve the availability of maternal and child health services. To explore the links between health facility electrification and service availability and use, the World Health Organization (WHO), in partnership with Dodowa Health Research Centre and Makerere University School of Public Health, carried out an implementation research study. The objectives of this study were to (1) capture changes in service availability and readiness, (2) describe changes in community satisfaction and use and (3) examine the implementation factors of sustainable electrification that affect these changes. Data were collected through interviews with over 100 key informants, focus group discussions with over 800 community members and health facility assessment checklist adapted from the WHO’s Service Availability and Readiness Assessment tool. Implementation factors were organized using Normalization Process Theory constructs. The study found that access to energy is associated with increased availability of health services, access to communication technologies, appropriate storage of vaccines and medicines, enhanced health worker motivation and increased community satisfaction. Implementation factors associated with improved outcomes include stakeholder engagement activities to promote internalization, provision of materials and information to encourage participation, and establishment of relationships to support integration. Barriers to achieving outcomes are primarily health systems challenges—such as drug stockouts, lack of transportation and poor amenities—that continue to affect service availability, readiness and use, even where access to energy is available. However, through appropriate implementation and integration of sustainable electrification, strengthened energy infrastructure can be leveraged to catalyze investment in other components of functioning health systems. Improving access to energy in health facilities is, therefore, necessary but not sufficient for strengthening health systems.

**KEY MESSAGES**

- Improved energy access in rural health facilities is associated with increased availability of services (particularly at night), appropriate storage of vaccines and medicines, and health workers’ self-assessed ability to carry out maternal and child health-related tasks.
- Availability of sustainable energy in rural health facilities is associated with improved community satisfaction of available health services.
- Early and continuous engagement of stakeholders at the national, regional and local levels is required to ensure the suitable installation of energy infrastructure, community buy-in and participation, positive knock-on effects, and sustainability.
- Improving access to energy in health facilities is necessary, but not sufficient, for strengthening health systems and can support the strengthening of other health system components such as workforce retention, access to medicines and availability of essential equipment.

## Introduction

Many primary healthcare facilities in low- and middle-income countries do not have adequate access to electricity. A 2013 review of nationally representative data in 11 countries in sub-Saharan Africa found that, on average, one in four healthcare facilities did not have energy access (Adair-Rohani *et al.*, 2013). Only 28% of healthcare facilities were found to have reliable (defined as not having prolonged interruptions) access to electricity (Adair-Rohani *et al.*, 2013). This energy gap has clear implications on the functionality of healthcare facilities and the quality, availability and safety of essential health services delivered within them (Franco *et al.*, 2017).

The World Health Organization’s (WHO) Service Availability and Readiness Assessment (SARA) tool identifies access to energy as a core requirement of functional health facilities, essential for the operation of basic amenities, including lighting, refrigeration, sterilization, ventilation, communications and computer systems (WHO, 2015b). It is also required for the safe management of medical waste (e.g. non-incineration methods) and access to clean water, as well as for the operation of essential medical devices, including emergency surgical, laboratory and diagnostic equipment (WHO, 2015a). Energy security affects other health system elements such as attraction and retention of the health workforce, health facility hours of operation and health service costs (WHO, 2015a). Scaling up sustainable energy infrastructure in health facilities not only improves service availability and quality but also contributes to ‘greening’ the health sector by relying on cleaner and more resilient options (Haines *et al.*, 2007; Franco *et al.*, 2017).

Despite these benefits, strengthening energy infrastructure in rural areas remains a challenge due to the lack of planning, maintenance, community participation, sustainable financing and institutional adaptability (Bhattacharyya, 2013; Feron, 2016). Improved understanding of what strategies help to mitigate these challenges is needed to help reduce health disparities caused by poor energy security.

### The intervention

In 2015–19, the United Nations Foundation (UNF), with funding from the UK Department for International Development, supported the ‘Powering Healthcare’ programme in deploying solar energy solutions to rural health facilities in Ghana and Uganda. The intervention was implemented under the auspices of ‘Energy for Women’s and Children’s Health’ and sought to enhance access to reliable electricity for the delivery of maternal and child health services. To ensure the successful implementation and sustainability of the project, Powering Healthcare and UNF engaged multiple local and international partners across both the health and energy sectors.

At the national levels, implementation partners included: All in Trade Ltd. in Uganda and Power World Ltd. in Ghana—both supported by the Solar Electric Light Fund. African Solar Designs Ltd., a Kenya-based renewable energy company, provided an initial needs assessment that informed the intervention. Ghana’s Ministry of Energy and Uganda’s Rural Electrification Agency were consulted and engaged in ensuring alignment with current and future government-led electrification plans. Stakeholders at different levels of the health system were engaged through Ghana Health Service and Uganda’s Ministry of Health to define service delivery needs and support the integration of energy infrastructure in health facilities.

Electricity needs can be categorized into ambient lighting (general lighting for the facility and health workers), task (lighting for special tasks and electricity to power essential equipment) and security (outdoor lighting for safety) (UNF; ASD, 2015a,b). African Solar Designs’ needs assessments found that decentralized energy infrastructure—particularly small solar photovoltaic systems—provides critical but insufficient energy access across all three categories (UNF; ASD, 2015a,b). They determined that the quality and reliability of energy infrastructure vary from service to service but are generally inadequate for maternal and child health services (UNF; ASD, 2015a,b). Furthermore, they found that many rural health facilities had pre-existing solar photovoltaic systems that were no longer maintained or operational due to poor integration, sustainability and scale up (UNF; ASD, 2015a,b). The key challenges identified were the low functionality of systems, lack of community ownership and no ongoing investment in the maintenance of the systems (UNF; ASD, 2015a,b).

Powering Healthcare and UNF aimed to avoid these pitfalls through following recommendations made in the needs assessments and by national partners. They planned the installation of solar photovoltaic systems based on anticipated future energy load requirements rather than limiting system capacities to current needs, allowing for further potential investment in health facility capabilities. They planned community and stakeholder engagement activities targeted to different stakeholder groups and at various stages of implementation. And they worked with national implementers who have the skills and expertise necessary to monitor and maintain solar photovoltaic systems remotely. In doing all this, the goal of the intervention was the sustainable implementation of reliable energy infrastructure in rural health facilities to improve the delivery of maternal and child health services.

### Study rationale and objectives

For the intervention to achieve its goal, the integration of solar photovoltaic systems (electricity) into the daily operations and contexts of rural health facilities and the adoption of energy-dependent equipment are critical. Therefore, to understand the outcomes of the intervention, the implementation factors affecting the integration and adoption of energy capabilities must also be examined. To study the outcomes and implementation of Powering Healthcare’s approach to solar electrification of rural health facilities, UNF partnered with the WHO’s Alliance for Health Policy and Systems Research and the Department of Public Health, Environmental, and Social determinants of health. The objectives of the study were

1. To explore changes in service availability and readiness following solar electrification of rural health facilities;
2. To describe whether electrification of health facilities affects demand for, utilization of and satisfaction with maternal and child health services; and
3. To examine the effect of implementation factors on the adoption and integration of improved energy capabilities and their roles in achieving service availability, readiness and use outcomes.

### Study context

#### Ghana

Seventy-nine percent of the general Ghanaian population has access to electricity, with regional and geographical variations (Bhattacharyya, 2013; IRENA; IEA; WHO; WB, 2019a). That is, while 78% of urban dwellers are connected to the national grid, <30% of rural dwellers have access to electricity (Cosgrove-Davies and Moussa, 2019). Ghana’s health system includes three administrative levels: national, regional and district (including sub-district and community) (UNF; ASD, 2015a). The operationalization of Ghana Health Service’s Community-based Health Planning and Services (CHPS) Policy has been a critical element of reform in improving access to health care by establishing CHPS compounds to deliver basic services to under-served populations at the village and community levels. In this study, the focus is on sub-district and community-level primary healthcare centres—often CHPS compounds—as they are least likely to have access to electricity and are the point of access to health services for a large portion of the population (UNF; ASD, 2015a). Services available at these levels include preventative, informational, maternal and child health, outreach and referral (Ghana Health Service, 2002; Johnson *et al.*, 2015).

**Figure 1:**
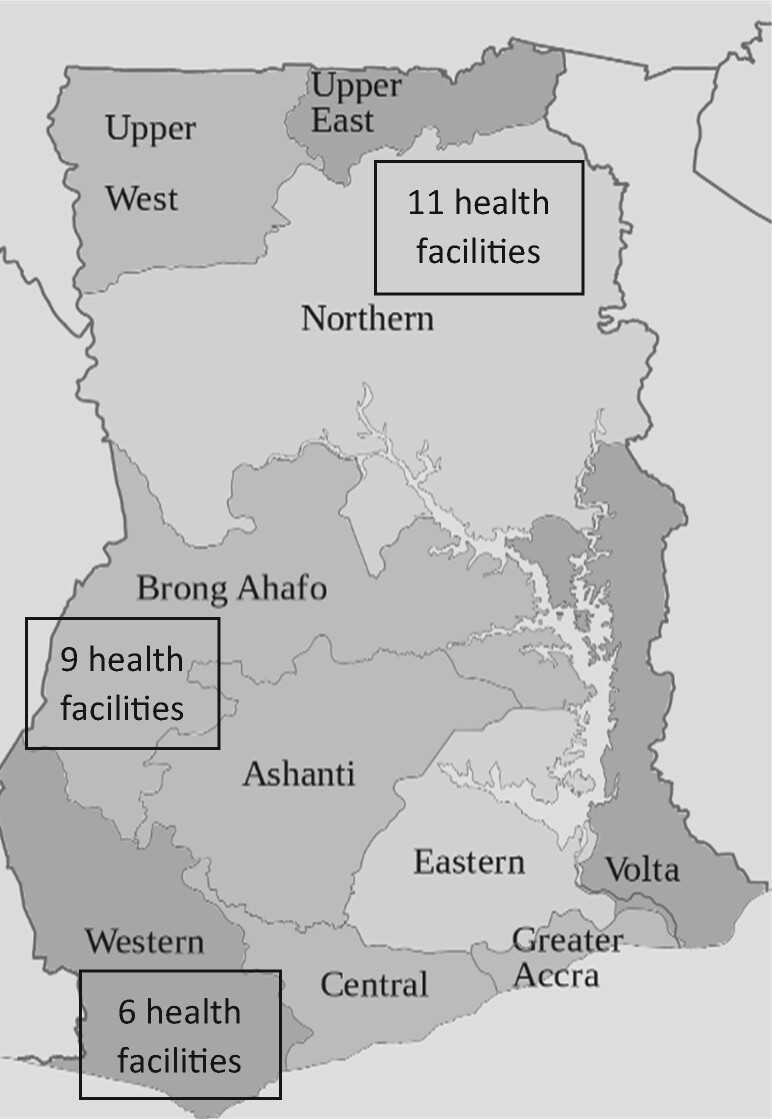
Map of Ghana showing study regions.

During a needs assessment to inform the selection of health facilities for electrification, African Solar Designs carried out 75 audits in six regions across Ghana, 87% of which were on CHPS compounds (UNF; ASD, 2015a). They assessed both energy demand and energy supply (UNF; ASD, 2015a). Based on their audit, priority was given to sites with no existing electrical power, longest distance from the national grid (and therefore lower potential of being connected in the future) and serving a larger population (UNF; ASD, 2015a). Electrification activities were implemented in 26 health facilities across three regions: Brong Ahafo Region (middle), Northern region (northern savannah) and Western region (coastal) reflecting the three ecological zones of the country (see Figure 1).

#### Uganda

Uganda has one of the lowest electricity consumption rates in Africa, with the electrification rate at 27% nationally and 18% in rural areas where ∼83.6% of the population resides (IRENA; IEA; WHO; WB, 2019b). In 2012, the government rolled out the Rural Electricity Strategy Plan to attain a 22% rural electrification rate by 2022 and universal access by 2035 (Rural Electrification Agency, 2013).

Health governance in Uganda is decentralized and split across national and district levels. Health centre I represents the village level, offering preventative and health promotion services (UNF; ASD, 2015b). Health centre II represents the parish and offer outpatient services, antenatal care, emergency deliveries and immunizations (Namazzi *et al.*, 2015). Health centre III represents the sub-county and offers acute admissions, maternity and basic laboratory services (Mijumbi-Deve *et al.*, 2017).

**Figure 2:**
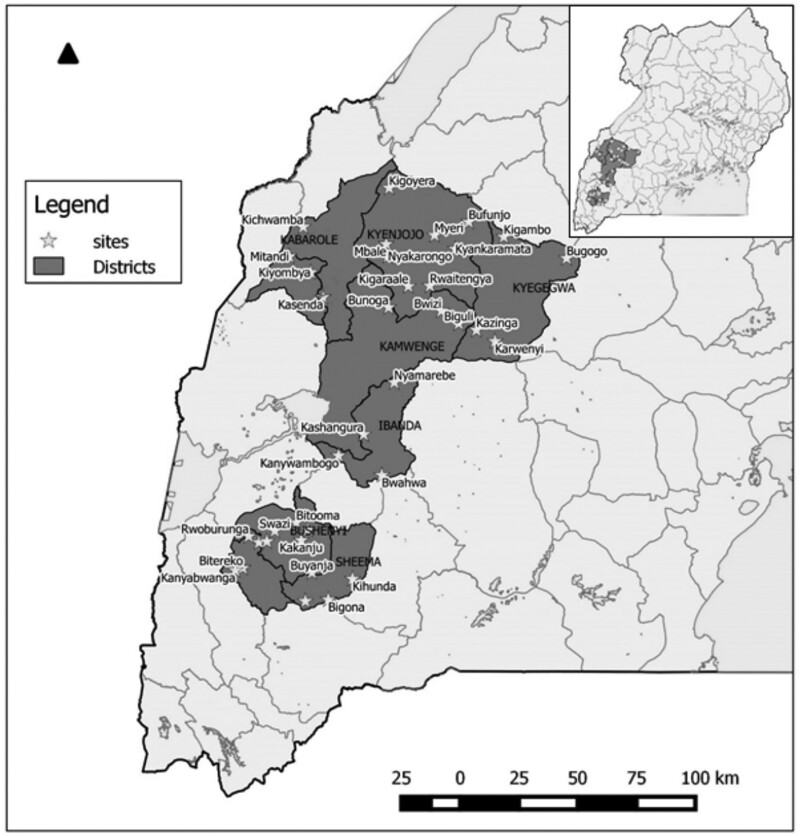
Map of Uganda showing study sites.

During the needs assessment conducted in Uganda, African Solar Designs consulted 100 health facilities—48% of which were health centre II and 42% health centre III—to assess energy demand and supply . Based on the audit, priority was given to facilities that were off-grid and government-operated to ensure ownership and sustainability, located in priority districts with large populations, and providing maternal and child health services (UNF; ASD, 2015b). Thirty-six facilities across eight districts in the western region were selected for electrification—all of which were health centre II or III (see Figure 2).

## Methods

**Figure 3:**
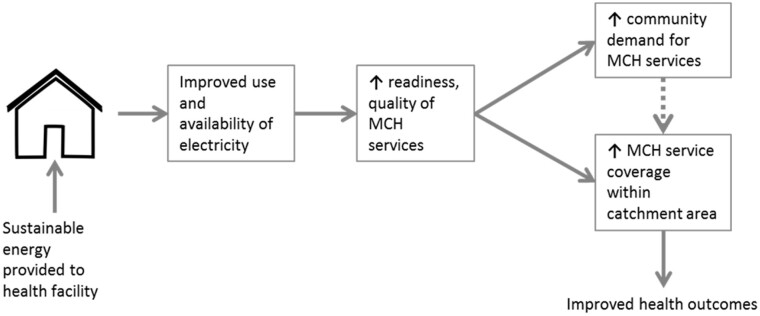
Theory of change.

The theory of change for the intervention asserts that the availability of reliable electricity would improve the availability, readiness^1^ and quality^2^ of essential maternal and child health services provided in rural health facilities, thereby contributing to the increased use of health services (see Figure 3). To capture measurable changes in service availability and readiness (objective 1), an adapted version of the WHO’s SARA tool in the form of a health facility checklist was used. The SARA tool is a health facility assessment tool used to generate information on the availability of crucial infrastructure, staff, basic equipment, amenities, essential medicines and the capacity of health facilities to deliver services (WHO, 2015b). For this study, items relevant to energy infrastructure, primary care and maternal and child health services were selected and used as an adapted health facility checklist.

To describe changes in community demand, use and satisfaction (objective 2), health facility records on attendance and focus group discussions (FGDs) around user perceptions and satisfaction with health facility infrastructure and service delivery were used.

Finally, Normalization Process Theory (NPT) constructs were applied to examine the role of implementation factors in the adoption and integration of improved energy capabilities and their role in whether (or not) changes to service availability, readiness and use take place (objective 3). NPT is concerned with implementation (the original plans and fidelity to these), embedding (when behaviours and use become routine) and integration (when the intervention is institutionalized) (May and Finch, 2009). NPT postulates that four processes are involved in ensuring that an intervention is adopted and sustainably integrated (i.e. normalized) into a system. Table 1 outlines the four NPT constructs, definitions and relevant implementation factors. These constructs were used in the development and analysis of interview and FGD questions concerned with implementation.

**Table 1:** NPT constructs and related implementation factors

NPT construct	Definition	Related implementation factors
Collective sense-making	Promoting coherence of a practice by users	Stimulating stakeholder awareness and understanding of changes expected through a practice (differentiation) Ensuring coherence across users in terms of the value of a practice (internalization)
Cognitive participation	Users’ legitimization and adaptation of a practice	Stimulating individual and collective motivation for participation (initiation) Defining roles and differentiating responsibilities (enrolment) Generating buy-in based on existing desires and contextual realities (legitimation) Providing materials and information necessary to carry out roles (activation)
Collective action	Supportive or inhibitory actions by users to enact a practice	Managing users’ interpretations of roles and their interactions with one another in action (interactional workability) Integration of a practice based on users’ skills and actions (skill-set workability) Monitoring operationalization of a practice in relation to existing structures and procedures (contextual integration)
Reflexive monitoring	Enhanced understanding of the effects of a practice	Ongoing assessment of the process, value and impact (communal appraisal) Adjustment and adaptation to enhance impact (reconfiguration)

Adapted from ‘Implementing, Embedding, and Integrating Practices: An Outline of Normalization Process Theory’ (May and Finch, 2009).

### Study design and data collection tools

This was a multi-country case study using mixed qualitative and quantitative methods to study the outcomes and implementation process of a rural health facility electrification programme 1-year post-installation. The in-country study was carried out by local research teams at Makerere University School of Public Health in Uganda and Dodowa Health Research Centre in Ghana. Research teams included research assistants, translators with research training for local languages and principal investigators. Research teams were not connected to the villages in which the research was conducted and had no source of pre-existing bias. Methods of data collection included key informant interviews, FGDs, administration of a health facility checklist, a review of facility records and administration of semi-structured questionnaire interviews. All data collection tools were tested during a training workshop in both Uganda and Ghana for the research assistants. Suggested prompts, based on NPT constructs, were included in semi-structured interviews. All interviews and FGDs were audio-recorded and supplemented by hand-written notes. Table 2 summarizes data collection tools used for each of the study’s three objectives at both baseline and endline 1 year later.

**Table 2:** Objectives and data collection tools

Objectives	Data collection tools	Information provided
1. To explore changes in service availability and readiness following solar electrification of rural health facilities	Health facility checklist	Changes in availability of essential equipment, basic amenities, services provided
	Semi-structured interviews with health facility staff	Health worker motivation and self-efficacy in delivering services
	Key informant interviews with implementers, district health officers, etc.	Changes in resource allocation and availability from the district level
	FGDs with community members	Observed changes in health facility infrastructure
2. To describe whether electrification of health facilities affects demand for, utilization of and satisfaction with maternal and child health services	Health facility records on attendance	Utilization of health services (antenatal care and deliveries)
	FGD with community members	Use patterns and satisfaction with health services
3. To examine the effect of implementation factors on the adoption and integration of improved energy capabilities and their roles in achieving service availability, readiness and use outcomes	Key informant interviews with implementers, district health officers, etc.	Implementation factors contributing to successes and challenges, organized through NPT constructs
	Semi-structured interviews with health facility staff	Assessing sense-making and cognitive participation (e.g. did staff understand the goal of the intervention and their roles)
	FGDs with community members	Assessing sense-making, cognitive participation and collective action

### Data sampling

In Uganda, from the 36 health facilities electrified, a randomly selected group of 25 Health facilities in the Kyegegwa, Kabarole, Kyenjojo, Kamwenge, Ibanda, Sheema, Bushenyi and Mitooma districts were included for study. These were selected using computer-generated identification numbers for each facility and random sampling. In Ghana, all 26 electrified primary health facilities—categorized as CHPS compounds—were included in the study.

In both countries, community members included in the FGDs were selected using convenience and snowball sampling. Selection criteria for FGD discussants included: a pregnant or postnatal woman and/or caregiver of a child under the age of 5, who had sought maternal and child health services from the selected healthcare facility within a year, and living within 5 km of the facility. Participants were identified and recruited during antenatal care visit days at the health facilities, through referral from community members, and through self-selection. FGDs were conducted in a private setting and spanned an average of 1 h. All available health workers were recruited for semi-structured interviews lasting 1–1.5 h and taking place in a private room at the health facility. Health facility checklists were completed through observation and with input from health workers. Key informants were purposively sampled based on their roles and connections with the implementation of the project. They included district and local leaders, ministry staff, engineers and implementing partners. Interviews spanned ∼1 h and were conducted in person for most participants except those not living in the countries in which the study took place. Different individuals were sampled at baseline and endline due to the turnover of key informants and availability of community members; however, the stakeholder groups from which they were sampled remained consistent.

In Uganda, a total of 432 and 500 community members seeking maternal and child health services participated in 25 FGDs at baseline and endline phases of the study, respectively. A total of 25 healthcare providers—one available at each facility—were interviewed using the semi-structured questionnaire, and 50 key informant interviews were conducted at baseline and endline. In Ghana, a total of 488 and 399 community members seeking maternal and child health services participated in 29 FGDs at baseline and endline phases of the study, respectively. A total of 45 and 36 health workers were available for interviews at baseline and endline phases, respectively, and 22 key informant interviews were conducted.

### Data analysis

Quantitative data (health facility checklists, health facility records) were analysed using STATA 14. Qualitative data (key informant interviews, semi-structured questionnaires and FGDs) were analysed using the thematic content analysis. All FGDs and key informant interviews were transcribed verbatim and analysed using ATLAS.ti.8.0. Two research team members read the transcripts several times and independently developed codebooks using elements of service availability, readiness, use, satisfaction and NPT constructs described above to guide the process. The members discussed any issues and agreed on a final codebook. The transcripts were coded and categorized into emerging themes and sub-themes. Preliminary results were discussed with multiple stakeholders, and final analysis was reviewed and refined based on feedback from these discussions.

## Results

### Baseline situation

The baseline health facility assessment in Ghana painted a picture of understaffed facilities with low capacity for delivering maternal and child health due to a lack of amenities, communication tools and equipment such as vaccine refrigerators and foetal dopplers. Only 2 out of the 26 facilities provided services 24 h a day for 7 days. Only 6 facilities had a midwife on staff, though 12 provided delivery services.

In Uganda, the baseline assessment showed a similar staffing situation; however, more midwives were available than in Ghana, and clinical officers were also posted at health centres II and III as these operate differently to Ghana’s CHPS, which are more reliant on community health nurses. In Uganda, there were slightly higher readiness capabilities as more facilities had access to some form of electricity, albeit unreliable, as well as equipment such as vaccine refrigerators and autoclaves.

### Changes in service availability and readiness

Administration of the health facility checklist in Ghana revealed statistically significant changes in the availability of 24-h emergency services, access to a mobile communication device supported by the facility, access to a reliable source of electricity with no interruptions and availability of energy-dependent equipment such as vaccine refrigerators (see Table 3). In Uganda, fewer significant changes were observed—in part due to existing equipment and electricity at baseline. However, the functionality and reliability of these amenities and equipment were presented as challenges by health workers. A statistically significant change in the reliability of electricity with no interruptions was observed at endline (see Table 4). This change in reliability could also lead to reviving unused equipment. For example, idle equipment observed by study teams, such as microscopes and vaccine refrigerators, could be converted to solar power. In both countries, changes to basic amenities, including access to clean water, sanitation facilities, safe disposal, computers and internet, were not observed.

**Table 3:** Availability of communication systems and power at health facilities in Ghana

		Period	
		Baseline *N* = 26 facilities (%)	Endline *N* = 23 facilities (%)
Communication systems at the health facility			
Offers 24-h emergency services	Yes	2 (7.7)	23 (100.0)*
	No	24 (92.3)	0
Functioning cellular telephone or a private cellular phone that is supported by the facility	Yes	0	18 (78.3)*
	No	26 (100)	5 (21.7)
Have a functioning computer	Yes	4 (15.4)	2 (8.7)
	No	22 (84.6)	21 (91.3)
Access to email or internet within the facility	Yes	5 (21.7)	5 (21.7)
	No	17 (65.4)	18 (78.3)
Power supply			
Availability of electricity at the health facility	Yes	14 (53.9)	23 (100.0)*
	No	12 (46.2)	0
Reliability of electricity	Always available with no Interruptions	0	21 (91.3)*
	Often with some interruptions (interruptions <2h)	10 (38.5)	1 (4.4)
Energy-dependent equipment	Vaccine fridge (Solar)	1 (3.8)	11 (47.8)*

* *P*-value <0.05.

**Table 4:** Availability of communication systems and power at health facilities in Uganda

		Period	
		Baseline *N* = 25 facilities (%)	Endline *N* = 25 facilities (%)
Communication systems at the health facility			
Offers 24-h emergency services	Yes	9 (36)	10 (40)
	No	16 (64)	15 (60)
Functioning cellular telephone or a private cellular phone supported by the facility	Yes	5 (20)	6 (24)
	No	20 (80)	19 (76)
Have a functioning computer	Yes	4 (16)	8 (32)
	No	21(84)	17 (68)
Access to email or internet within the facility	Yes	0 (0)	4 (16)
	No	25 (100)	21 (84)
Power supply			
Availability of electricity at the health facility	Yes	24 (96)	25 (100)
	No	1 (4)	0 (0)
Reliability of electricity	Always available with no Interruptions	13 (52)	25 (100)*
	Often with some interruptions (interruptions <2h)	4 (16)	0 (0)
	Sometimes available (interruptions >2h)	8 (32)	0 (0)
Energy-dependent equipment	Vaccine fridge (solar)	25 (100)	25 (100)

* *P*-value <0.05.

Semi-structured interviews with health workers and FGDs with community members revealed potential for upcoming changes to service availability and readiness. For example, study teams in both countries noted plans by community members and by local decision-makers to build additional structures, including operating theatres, wells and housing for health facility staff (see Table 5, ‘Infrastructure enhancements’).

**Table 5:** Illustrative quotes on themes identified in interviews and FGDs

Elements	Supporting quotes
Infrastructure enhancement	*The provision of the solar panels has indeed impacted positively on the three health facilities. For community members in Soo are now more willing to support the health sector in finding decent accommodation for staff who accept posting to their community. In Soo [CHPs], for example, a midwife has accepted posting to the place. As soon as community members heard this information, they have volunteered to build a small staff accommodation for the midwife to make her stay more comfortable* (Health staff, Ghana). *You see a hole that has been dug close by (pointing the research team to the dugout pit)? Measures have been taken to dig a well to supply water to them (staff) up there by tapping into the solar energy to pump water to the facility when it is done. […]. The MP of the area is the one who has decided to do that for the facility. This would improve water situation in the facility to do their work and relieve mothers the stress of providing their own water when they seek healthcare, especially during delivery service […]* (Community member, Aowin district, Ghana). *The electrification of this health facility has opened doors for the construction of a theatre, ward and other equipment in the future. In addition, someone can even invest in a canteen to provide cold drink to patients* (FGD participant, Swazi health centre II, Uganda).
Resource and equipment allocation	*[…] Now they are calling for more equipment … you see we use the steam broiler now they say they want autoclave. I asked them if the solar can provide the power for the autoclave. So, we went to ask for the price of a small one for them, so that it does not consume a lot of energy […]* (District Director, Ghana). *Districts and other facilities can now start planning for equipment like centrifuges, oxygen, etc. Even partners may be willing to provide equipment because they know they will be put to use since there is adequate energy* (Key informant, MoH, Uganda).
Health worker quality of life	*In the days when the facility had no electricity, they [providers] did not have any form of entertainment. It was one of the reasons they decided not to be at post most of the time…. For some of them, their spouses have joined them because the facility now has electricity […]* (Health worker, Northern region, Ghana). *Accommodation is bad, because all the staff don’t have accommodation. And the other health workers house has a very big crack. You can even see it there on the side, it has a very big crack, almost breaking away* (Health team member, Uganda). *In fact, a positive thing I have seen with the coming of the solar is that now they (providers) are more stationary in the facility […] because they can now charge their phones in the facility and to communicate with their families. Therefore, they no longer travel to the city, often leaving the facility unattended, because they had to charge their phones. […] means that they are now happy to stay there [facility] and work* (District Director, Ghana). *Lighting has really improved service provision. The provision of electricity made life of health workers easy and gave them the confidence to come and work here. It has also boosted the confidence of the sick people in the health facility. These factors have, in turn, increased the number of patients accessing care from here* (Local council chairperson, Ibanda district, Uganda).
Community trust and satisfaction with health workers	*[…] The providers have stopped their behaviors of refusing to wake up at night to attend to you or asking you to call the security man who might be sleeping or might not have come to work due to ill health to confirm your identity to them […] These days when you knock on their door at night, they would immediately look through the window if they identify you as a community member immediately they come out to attend to you* (Community member, Ghana). *… In the past when Kpanashie [healthcare facility] did not have electricity, the healthcare providers stored their vaccines and medicines in the nearest community and picked them up when they needed them. So, when you go there to vaccinate your child, they would tell you to return for the vaccination another time. ‘[…] Now with that the solar, they [health worker] have been given a fridge to store their drugs. So, when you take the child there for vaccination, they don’t turn the child away* (Community member, Ghana). *The services related to maternal health at this facility started improving following the coming of this solar because, you would find the health worker going to help a mother deliver lighting using a torch but when the solar became available the services have improved and more women are coming to the health facility. The midwives that are here and help the mothers a lot when the solar services was installed at the facilities, the people have benefited by not traveling long distances looking for other health facilities* (Village health team, Ibanda district).
Community trust and satisfaction with health services	*The electricity is important. Some time ago my child was admitted at Mampong the drip on him was not finished because there was no light. I was even afraid of the unknown when I was sleeping there. So, I told the nurses to discharge us. I went home to sleep and sent him back for care again the next morning. If the light had been there, like it is now, I could have slept there […]* (Mother, Ghana). *I am attracted to come back to this health care facility since health workers no longer use torches to deliver mothers due to availability of electricity. Availability of energy has provided enough light to us as mothers to have safe deliveries* (Mother, Uganda).
Health system barriers	*Medications are very essential. When you go to the ‘doctor,’ you know they have to give you medication. But you would go there and still come home to buy all the medications, so the medicines are our problem, the solar has not changed the situation* (Community member, Ghana). *The other challenge is that the facility is small, especially the maternity ward. The room is so small, congested. If women come to deliver and they are 2 or 3, they can’t find where to deliver from. It is a small facility. And the midwife cannot do anything, so she has to get one off the bed and put on another one. That is one of the biggest challenges in the maternal services. And that makes some of them to go to other places* (Village Health Team Member, Uganda). *Sustainable energy will improve service delivery, greatly of course when staffing and drugs are also enough, if we had enough staff, enough drugs, and then electricity, then water, and transport!* (HFMC chairman, Uganda). *You come to the facility, and they tell you that there are no drugs; you are pregnant, and you have to move and go and look for the drugs. You don’t even find a single drug. A month passes and you come, second month and come back, but there is no drug available all this time. So, what do we do?* (Community Member, Uganda).
Electricity as a catalyst for strengthening access to medicines and medical devices	*In the past when they did not have electricity, the providers had to come all the way here (the DHMT) to pick their vaccines for the day in order to provide immunizations at CWC (child Welfare Clinic) and also vaccinate pregnant women. They also had to return any unused vaccines at the end of the day because they had no fridges in the facilities. Now that they have the solar, they have been provided with small fridges to enable them keep vaccines in their facilities. This has minimized the need to travel all the way to District (District Health Administration) for all these reasons* (DHMT member, Ghana). *We used to keep our drugs in Ishongolo (nearby health facility with hydroelectricity), but we now have a fridge. Therefore, we are able to keep drugs. We are now told by the health care providers that drugs, which require to be kept in cold conditions are now stored well because of the electricity* (Local leader, Uganda).

Similarly, key informant interviews with local and regional decision-makers in both countries revealed changes in resource allocation plans. This suggests that the availability of electricity at health facilities made facilities more likely to receive basic energy-dependent equipment such as autoclaves, centrifuges and vaccine fridges. Study teams also observed that after electrification, health facility staff were more likely to demand equipment from district leaders due to the improved energy infrastructure and capacity (see Table 5, ‘Resource and equipment allocation’).

In addition to physical infrastructure and equipment, another important component of service availability and readiness is the availability of a trained health workforce (WHO, 2015b). At baseline, interviewed health workers and community members in FGDs expressed challenges in terms of health worker availability, motivation, retention and attitudes. Some health workers were described as having bad attitudes, low motivation and poor living conditions (see Table 5, ‘Health Worker Quality of Life’).

In this study, no significant changes were observed in the number of health workers available. However, changes in satisfaction and self-assessed ability to carry out tasks were articulated in health worker interviews. In Ghana, there was a statistically significant change in health workers’ satisfaction with electricity services at the health facility. All 36 (100%) of those interviewed shared that since electrification, they have had adequate lighting to conduct tasks in the maternity department both during the day and at night—a change from 1 (2%) expressing the same at baseline (see Table 6). In Uganda, similarly, a statistically significant change in provider satisfaction was observed, with 24 (96%) of those interviewed stating that they had adequate lighting to conduct tasks both during the day and at night—a change from 2 (9%) expressing the same at baseline (see Table 7).

**Table 6:** Provider satisfaction in Ghana

Indicator		Period	
		Baseline, *N* = 45 (%)	Endline, *N* = 36 (%)
Satisfaction with electricity services at the health facility	Yes	0	36 (100.00)*
	No	42 (100.00)	0
Have adequate lighting for conducting day tasks in the maternity department	Always	0	36 (100.00)*
	Sometimes	0	0
	Never	0	0
Have adequate lighting to perform night tasks in the maternity department	Always	1 (2.22)	36 (100.00)*
	Sometimes	18 (40.00)	0
	Never	24 (53.33)	0
	Not applicable	2 (4.44)	0

* *P*-value <0.05.

**Table 7:** Provider satisfaction in Uganda

Indicator		Period	
		Baseline, *N* = 24 (%)	Endline, *N* = 25 (%)
Satisfaction with electricity services at the health facility	Yes	0 (0)	19 (76)*
	No	24 (100)	6 (24)
Have adequate lighting for conducting day tasks in the maternity department	Always	16 (66.7)	24 (96)
	Sometimes	5 (20.8)	0 (0)
	Never	3 (12.5)	1 (4)
Have adequate lighting to perform night tasks in the maternity department (*N* = 22 at baseline, *N* = 23 at endline)	Always	2 (9.1)	22 (95.7)*
	Sometimes	9 (40.9)	1 (4.3)
	Never	11 (50)	0 (0)

* *P*-value <0.05.

Emergent themes from health worker interviews, key informant interviews with district leaders, and FGDs with community members include changes in access to health workers, improvements in the quality of care they can deliver and an enhanced overall quality of life for staff. Health workers frequently reflected on improvements to their living situation. District leaders emphasized improved health worker perceptions towards being posted at newly electrified health facilities as well as full-time accessibility of health workers within the villages (see Table 5, ‘Health Worker Quality of Life’).

FGDs with community members supported these claims and reflected improved user satisfaction with the quality and availability of health services delivered by health workers (see Table 5, ‘Community trust and satisfaction with health workers’).

### Changes in use of and satisfaction with services

Community members’ satisfaction with health facilities saw a statistically significant improvement in both countries following electrification. In Ghana, 47 (9.6%) of 488 community members claimed they were satisfied at baseline while 378 (94.7%) of 399 said the same at endline. In Uganda, 147 (34.0%) of 432 community members claimed they were satisfied at baseline, while 477 (95.4%) of 500 said the same at endline.

Emergent themes around the satisfaction of community members with health facilities include use of the health facility at night, improved perceptions around safety, cleanliness and quality of care and a minor theme around pride and trust towards the health facility as a result of its improved infrastructure (see Table 5, ‘Community trust and satisfaction with health services’).

Despite improved satisfaction and perceptions of community members towards quality and availability of services at the health facilities, statistically significant changes in the use of services, measured by average number of deliveries and antenatal care visits, were not observed through review of health facility records at baseline and endline. Part of this may be explained by other barriers to accessibility. For example, in FGDs at both baseline and endline, community members highlighted transportation as the main challenge in accessing health facilities. Key informant interviews with district leaders and health workers also pointed to other health system elements affecting use of services. Emergent themes around health system barriers affecting demand and use include low availability of medicines and devices, size and privacy of health facilities, poor transportation, weak referral networks, poor water and sanitation services and insufficient human resources (see Table 5, ‘Health System Barriers’).

The introduction of solar electrification has started to address some of these barriers. For example, access to functional refrigerators has changed the availability of medicines and vaccines in some of the health facilities, but this is not yet widespread across all facilities as the new mode of operation takes time to normalize (see Table 5, ‘Electricity as a catalyst for strengthening access to medicines and medical devices’).

### Implementation, adoption and integration

NPT constructs were used to examine factors leading to the integration of electricity into the daily operations of rural health facilities and the adoption of energy-dependent equipment at district and community levels. This section identifies implementation factors, as relevant to each NPT construct (refer to Table 1), and discusses their roles in service availability, readiness and use outcomes described in previous sections.

#### Collective sense-making of sustainable solar energy

Sense-making across stakeholder groups is critical in establishing a coherent approach to adopting and using new technologies to achieve expected outcomes (May and Finch, 2009). In this intervention, stakeholders included leaders in the health and energy sectors, health workers, implementing partners and community members. Engagement and mobilization activities were done through continuous consultation, institutional partnerships, identification of point persons for communication between stakeholder groups and community mobilization events before and during installation. These efforts were made to set expectations for the project and the changes it would initiate, promote adoption of the new technology and create favourable attitudes and buy-in.

These efforts were largely successful, as reflected by the enthusiasm articulated in key informant interviews, health workers’ interviews and FGDs. By sensitizing district leaders and health workers to the potential for improvement and health system strengthening created by solar electrification, the implementers enhanced the likelihood of electricity use being embedded into daily practice beyond ambient lighting. For example, staff in some facilities began to use and transfer health management information systems using available communication tools (see Table 8.1).

**Table 8:** Illustrative quotes on NPT constructs

NPT construct	Supporting quotes
1. Collective sense-making	*The staff can now enter their own data on the DHMIS in the facility. So, as I sit here in my office now, if I turn on my laptop and at a click of a button, I know what is happening over there (facility) and monitor them so far as they key in into the DHMIS* (District Director, Ghana).
2. Cognitive participation	*They should put regulations on how it will be used. And tell people its use. And maybe they should give us people who trained in that area to come and educate us and the nurses. So that we know how to keep it well and safe* (Community member, Uganda).
3. Collective action	*LCIII is the leader. Imagine, if you bring it and you don’t involve LCIII, what will happen, who will go talking about the project? He will advocate for its repair and maybe find someone who can repair it* (Mother, Uganda). *I make sure that I pass at the health facility every evening on my way home from the nearby town center. What I want to make sure is whether the health facility has been lighted up. Every member is concerned, and we all guard the facility* (Local leader, Uganda). *in a way client’s privacy too has also been compromised because of the impact of the community members charging their phones in the facility […] Some clients want their privacy but because of the charging, other community members may see them coming for care, especially family planning services, which are private…* (District Director, Ghana). *That would need a project committee to take care of it, and that would help to sustain it. There needs to be a sub-committee in the HFMC, including health workers and sub-county members, to ensure project maintenance* (HFMC chairman, Uganda).
4. Reflexive monitoring	*[…]. Even this PC I have in front of me is part of the things they gave us for expert training … it is internet based so that we can monitor while sitting here in the office what is happening over there in the health facilities in terms of electricity reliability* (Clinical Engineer, Ghana). *We have been monitoring to ensure that everything is working well. I have even noticed that one of the bulbs was showing a sign that it had developed a problem and so we called the contractor and told him. […] he said it is not a major problem and so they will be coming into the community in two months to check that* (Committee member, Ghana).

Given the significance of relationship building and long-term engagement in achieving outcomes, the high turnover rate of district and health facility staff observed by the study team is a barrier to sense-making and sustainability. Another challenge is that if expectations are not aligned across stakeholders and remain unmet, trust and, therefore, demand for the technology could be compromised. This study revealed that despite community mobilization events, community members articulated expectations beyond the scope of the project. They had expectations of advanced surgical services, recruitment of specialized health providers, and improved water supply for the villages. The lack of changes in the use of health facilities could be partially connected to these higher expectations not yet being fulfilled by the reality of available services.

#### Cognitive participation

Cognitive participation was assessed by exploring how the implementation of the intervention motivated health workers, implementing partners and the community to play an active role in maintaining sustainable energy systems and leveraging energy infrastructure to enhance service availability and readiness. Consultations with health workers during implementation focused on how electricity can improve readiness and their ability to deliver care if they work with district health officers to adopt energy-dependent equipment. They were also connected with national implementing partners responsible for systems maintenance so that they had someone to contact in the event of system failures. These efforts at the enrolment and activation of health workers were successful; health workers expressed appreciation that their roles in the intervention do not infringe on their primary role of providing health services. They focused instead on how to leverage the technology to deliver better care.

Community members, however, expressed frustration at the lack of autonomy in repairing and monitoring solar photovoltaic systems and shared a desire to receive training within the villages to play a more active role (see Table 8.2).

By design, and to leverage existing in-country expertise, national implementing partners in this project have ownership over the monitoring, repair and replacement of solar photovoltaic systems. This was done based on lessons learned from previous failed initiatives. A means of strengthening this design could be to provide some technical information to those interested during community mobilization activities to facilitate the identification of common system malfunctions. In some villages, health facility management committees have taken this role upon themselves and are working to integrate the monitoring of solar photovoltaic systems into their existing duties.

#### Collective action

The success of this intervention is dependent on how different stakeholders work together to integrate stronger energy infrastructure in rural health facilities. Implementation steps taken in this intervention to promote integration and prevent inhibitory actions include linking stakeholders, communicating roles through targeted engagement and collectively defining appropriate actions to enhance the impact of the technology. Themes related to collective action identified in FGDs and interviews include improvements in communication channels between different levels of the health system, the establishment of roles and accountability mechanisms across stakeholder groups, and challenges related to existing contextual barriers that inhibit integration or incentivize inhibitory actions.

Respondents described a willingness to adopt new ways of working together and establishing communication channels across stakeholder groups to ensure the integration and maintenance of solar photovoltaic systems. Local leaders and community members viewed themselves as active participants in protecting the new energy infrastructure and had willingly taken on the responsibility to report problems (see Table 8.3).

The rise in accountability extends to district leaders as well, creating interactional workability. Through implementation consultation activities, district leadership has been encouraged to allocate resources to health facilities based on their new energy capacity but within the limits of primary care policy. For example, district health officers have coordinated with facility health workers to meet the need for energy-dependent equipment. In turn, health workers are expected to effectively use this equipment and increase the range of services available to communities. In select health facilities, district leaders have also used the opportunity created by electrification to strengthen water and sanitation infrastructure as well as information technology capacities. However, without recruitment of additional staff, health workers expressed feeling unable to take the advantage of new information technology capabilities.

Similarly, barriers such as lack of access to energy in the surrounding villages also inhibit the appropriate use of health facilities’ energy infrastructure and negatively impact the use of health services. For example, district leaders and community members shared concerns around lowered privacy due to higher use of health facilities for energy access, particularly for charging cell phones.

Creative approaches to contextual integration are necessary to overcome these challenges. Existing mechanisms in villages can be adapted to manage the unintended consequences of solar electrification. For example, Health Facility Management Committees are charged with the stewardship of various facility properties against theft or other potential damages. Committee members can be called upon to find solutions as they already feel activated to participate in the integration and sustainment of solar electrification (see Table 8.3).

Other existing assets identified for collective action include civil society organizations and funding agencies who operate in the regions of the study. These partners are more willing to provide equipment to electrified facilities because they know that the equipment can be used. Uganda’s engagement of partners such as Baylor and UNICEF since the inception of the project is an example of how these relationships can be cultivated over time to achieve synergistic effects on health facility readiness.

#### Reflexive monitoring

Active monitoring and iterative adaptation are essential in ensuring the sustainability of this intervention and in driving up demand for facility-based maternal and child health services. In the previous sections, we have seen examples of how solar electrification can set off a chain of knock-on effects that impact health system resilience. A break in the efficacy and effectiveness of solar electrification can quickly reverse this chain and lose newly generated demand for facility-based maternal and child health services. To avoid this feedback loop, robust monitoring and accountability by stakeholders across the different levels of the health system are required.

This intervention employed the use of remote monitoring—an opportunity for real-time monitoring that facilitates fast response, feedback and mitigation of overloaded systems.

In addition to remote monitoring, empowered community committees are required to ensure accountability and trigger adjustment and reconfiguration as necessary. Maintaining communication channels between health facility management committees, health facilities, implementers and district leadership is crucial for the sustainability of this project (see Table 8.4).

In the 1-year timeframe of this study, communication has been improving as the different stakeholders continue to negotiate and test roles and responsibilities. The next few years will determine the success and sustainability of these connections.

## Discussion

The research team theorized that strengthening the infrastructure of rural health facilities by providing reliable electricity would improve service availability, readiness and, therefore, use of maternal and child health services. Over 1 year, improvements to certain aspects of service availability and readiness, such as access to communication devices, basic equipment and night-time services, were observed. A shift in health worker satisfaction, motivations and attitudes was also captured, suggesting a potential for increased recruitment and retention of skilled health workers. Community members displayed increased satisfaction with health facilities; however, significant changes in the use of maternal and child health services were not observed. All respondents voiced concerns around health system barriers that affected the access to and quality of health services.

The observed effects in this study are likely smaller than they would be if other health system elements are also strengthened. These include elements identified as essential by the WHO ‘Standards for Improving Quality of Maternal and Newborn Care in Health Facilities’, which requires that ‘the health facility has an appropriate physical environment, with adequate water, sanitation and energy supplies, medicines, supplies and equipment for routine maternal and newborn care and management of complications’ (WHO, 2016).

Implementation factors used in this intervention played critical roles in supporting the adoption and integration of strengthened energy infrastructure in rural health facilities and in catalyzing investment in health system components such as medicines, equipment and workforce. As seen in other health system strengthening initiatives, sustainable energy infrastructure is necessary, but not sufficient, in increasing health system resilience (Namazzi *et al.*, 2015; Sharma *et al.*, 2015). The solar photovoltaic configurations provided to facilities in this project intentionally overestimated facility requirements to account for future growth and investment in these facilities by communities, districts and regional leaders. If the solar photovoltaic systems are well-maintained and sustained, one can expect to see a snowballing effect of enhanced resilience at these facilities. The early phases of this effect were captured by the allocation of energy-dependent essential equipment to electrified health facilities in this study. In certain villages, upgraded energy infrastructure inspired community members to build housing infrastructure for health workers. This contrasts with community members expressing concern around health worker attitudes at baseline. Health providers working in resource-limited settings sometimes display negative attitudes and behaviours as a result of the stress and pressure they experience (Bohren *et al.*, 2015). Access to electricity and community efforts to improve staff housing are factors that can contribute to alleviating stress and improving health worker satisfaction, as observed in health worker interviews.

To achieve further impact in terms of availability, readiness and use of maternal and child health services—as well as knock-on effects in health systems strengthening—sustainable energy solutions must continue to be integrated into district health systems’ management structures and maintained. Through community mobilization efforts, targeted and asset-based activation strategies, and the establishment of long-term consultative relationships, this project ensured that stakeholders had internalized the value of access to energy, enrolled themselves in its use, and collaborated in integrating its practice into their respective contexts. However, challenges remain. For example, community members felt that they had not been given sufficient information to support the basic maintenance of the system.

Basic training of a small group of community members to serve as local solar experts could be one means of addressing this concern. In other settings, strengthening of local technical capacity has been identified as a means of better supporting sustainable electrification initiatives (Palit, 2013). However, a significant barrier here is that non-authorized individuals can only carry out a limited range of maintenance activities to avoid the risk of voiding the warranty on the main components of the solar system. Any natural product failure within the warranty period must be responded to by the national contractor. This centralized approach was taken intentionally to support sustainability and allow for scale up while still providing implementers with the flexibility to make context-specific adjustments.

Another threat to integration is the high turnover rates among district and health facility staff. The threat can be mitigated by establishing a workflow between community-driven Health Facility Management Committees, health workers, local implementing partners, district officers and regional health officials. This would allow the maintenance, use and reconfiguration of solar photovoltaic systems to be systematically integrated into the existing context instead of relying on a few key stakeholders. Linked stakeholder groups can more efficiently work together to continue to promote the appropriate use of solar photovoltaic systems and to leverage these systems to improve health services. Health workers can use their new energy infrastructure to demand more from district and regional leaders and to set higher expectations for their facilities. District officers can showcase electrified facilities as examples for the scale up of sustainable energy systems and mobilize donor or government funding. Health Facility Management Committees can form regional networks through local implementing partners for support with maintenance, repairs and reflexive monitoring.

### Similarities and differences across countries

The streamlined implementation approach created many parallels in the engagement process and outcomes observed across Ghana and Uganda. For example, community members and health workers had similar expectations before and similar levels of satisfaction after installation. Challenges identified were also similar, including health system barriers affecting service delivery, uncertainties about the maintenance, and turnover of staff.

A key difference was the expected service delivery capacities of the facilities. Uganda’s health centre III should have a maternity ward and laboratory services; therefore, they are expected to deliver more services and house more equipment than the CHPS facilities studied in Ghana (Mijumbi-Deve *et al.*, 2017). As a result, the approach to strengthening other aspects of health facilities in Uganda, such as advanced equipment and partnerships with civil society, was more systematic than in Ghana, where community-driven improvements were more organically observed (e.g. staff housing).

### Strengths and limitations

This study uses an adaptation of the WHO SARA tool, a validated and recommended standard tool for measuring health facility readiness and availability of services, to assess readiness. An implementation research approach allowed for real-time assessment of implementation activities and followed a systematic framework using NPT constructs. In the design of this study, local implementers and researchers established a direct line of communication to support reflexive monitoring and to feedback learnings from observations and interviews into implementation design adjustments (e.g. Placement of light switches or number of outlets). This process was successful in mitigating design flaws that may have been more difficult to correct later. It also allowed implementers to better understand community expectations so that they could clarify their messaging during check-in visits.

A major limitation of the study is that the period of observation post-implementation was too short at 1 year to demonstrate real change in outcomes and impact as it will take time for service delivery norms and community perceptions to change. In addition, many factors contribute to changing health outcomes, and energy is just one. The facilities selected were often lacking in many of the other elements of a strong health system, so it was difficult to measure and observe technical aspects of service delivery where necessary resources and technical equipment were unavailable.

## Conclusion

This study demonstrates that solar electrification of health facilities can be successfully implemented to achieve positive outcomes in service availability, community satisfaction and health facility readiness—specifically, communication capabilities, appropriate storage of vaccines and medicines and health worker motivation. The health system barriers identified in the study—such as drug stockouts, lack of transportation, limited human resources and poor amenities—demonstrate that improving access to energy is necessary but not sufficient for strengthening health systems. Through appropriate implementation and integration, energy infrastructure can be leveraged to stimulate ownership and investment in strengthening other components of well-functioning health systems. Implementation factors associated with improved outcomes include stakeholder engagement activities to promote the internalization of project aims (sense-making), provision of materials and information to encourage active participation (cognitive participation), and establishment of relationships to support integration and sustainment (collective action and reflexive monitoring).

Future research studies could explore the impact of solar electrification on more technical aspects of care provision, including the use of specific equipment, the load required and training needs. This can facilitate service-specific energy needs assessments. Furthermore, cost-effectiveness evaluations of sustainable energy solutions are worth pursuing to provide insight into the financing implications of implementing similar interventions and negotiating purchasing, installation and maintenance, roles across sectors.

This study has explored one comprehensive approach for bringing reliable energy supply to remote health facilities. The lessons learned here can help other governments, implementers and donors in designing similar interventions to strengthen health systems.

## Footnotes

1 Readiness is defined here as per WHO SARA and refers to the capacity of the health facility to provide specific services.

2 Quality is defined here as services having been provided according to National or WHO standards of care (e.g. WHO guidelines on essential RMNCAH Interventions). User (patient and health worker) perspectives on quality of services provided is also considered here.
